# The autophagy inducer SMER28 attenuates microtubule dynamics mediating neuroprotection

**DOI:** 10.1038/s41598-022-20563-3

**Published:** 2022-10-25

**Authors:** Marco Kirchenwitz, Stephanie Stahnke, Kyra Grunau, Lars Melcher, Marco van Ham, Klemens Rottner, Anika Steffen, Theresia E. B. Stradal

**Affiliations:** 1 Department of Cell Biology, Helmholtz Centre for Infection Research, Inhoffenstrasse 7, 38124 Braunschweig, Germany; 2 Division of Molecular Cell Biology, Zoological Institute, Technische Universität Braunschweig, Spielmannstrasse 7, 38106 Braunschweig, Germany; 3 Division of Cellular and Molecular Neurobiology, Technische Universität Braunschweig, Spielmannstrasse 7, 38106 Braunschweig, Germany; 4 Cellular Proteome Research, Helmholtz Centre for Infection Research, Inhoffenstrasse 7, 38124 Braunschweig, Germany

**Keywords:** Autophagy, Cytoskeleton, Microtubules, Cell signalling, Cell biology

## Abstract

SMER28 originated from a screen for small molecules that act as modulators of autophagy. SMER28 enhanced the clearance of autophagic substrates such as mutant huntingtin, which was additive to rapamycin-induced autophagy. Thus, SMER28 was established as a positive regulator of autophagy acting independently of the mTOR pathway, increasing autophagosome biosynthesis and attenuating mutant huntingtin-fragment toxicity in cellular- and fruit fly disease models, suggesting therapeutic potential. Despite many previous studies, molecular mechanisms mediating SMER28 activities and its direct targets have remained elusive. Here we analyzed the effects of SMER28 on cells and found that aside from autophagy induction, it significantly stabilizes microtubules and decelerates microtubule dynamics. Moreover, we report that SMER28 displays neurotrophic and neuroprotective effects at the cellular level by inducing neurite outgrowth and protecting from excitotoxin-induced axon degeneration. Finally, we compare the effects of SMER28 with other autophagy-inducing or microtubule-stabilizing drugs: whereas SMER28 and rapamycin both induce autophagy, the latter does not stabilize microtubules, and whereas both SMER28 and epothilone B stabilize microtubules, epothilone B does not stimulate autophagy. Thus, the effect of SMER28 on cells in general and neurons in particular is based on its unique spectrum of bioactivities distinct from other known microtubule-stabilizing or autophagy-inducing drugs.

## Introduction

Autophagy is a cellular recycling process. In its most prevalent form, macroautophagy, cytosolic components are sequestered in double-membraned autophagosomes and degraded upon fusion with the lysosomal compartment^1^. Therefore, it is at the core of cellular and organismic homeostasis. Consequently, autophagy plays a crucial role in neuronal cell survival and function under physiological and pathological conditions^2^. A growing number of findings suggests that neurodegeneration is associated with deviations in autophagic flux.

Along these lines, defects in autophagy have been linked to neuronal dysfunction and degeneration^3^. In affected neurons of several neurodegenerative diseases such as Alzheimer's disease (AD), Huntington's disease (HD) or Parkinson's disease (PD), autophagosomes accumulate abnormally in axons prior to cell death^4^. Formation, maintenance and function of the axon is tightly coupled to the microtubule (MT) cytoskeleton in neurons^5^. MTs serve as transport routes for organelles like mitochondria or endomembrane vesicles, for polarity signals and other molecules such as RNA in cells and particularly in axons. Directed transport is driven by the protein families of kinesins (+ end directed) or dyneins (- end or MTOC directed)^6^. MTs are polar, hollow tubules built of α- and β-tubulin dimers. MTs originate most commonly from γ-tubulin seeds in the MT organizing centre (MTOC) and less frequently from other cellular locations. They polymerize by addition of GTP-loaded αβ-tubulin dimers to the so-called MT plus ends, resulting in their growth towards the cell periphery. MTs have fundamental roles in many essential biological processes, including cell division and intracellular transport, and significant advances were made in the understanding of microtubule plus-end-tracking proteins (+ TIPs) such as end-binding protein 1 (EB1) or its binding partner CLIP-170^7,8^. Not surprisingly, + TIP components have also been reported to control MT polymerization e.g. through EB1 phosphorylation by ASK1^9^ or by CLIP-170 phosphorylation through JNK^10^, LRRK1^11^ or AMP-activated protein kinase (AMPK)^12,13^.

Depolymerization of MTs is very dynamic and also occurs stochastically from the plus-end by so-called microtubule catastrophe events^14^. MTs are built of initially GTP loaded αβ-tubulin dimers, which hydrolyze GTP to GDP shortly after incorporation into MTs. Further aging of the microtubule is associated with posttranslational modifications such as the proteolytic removal of a C-terminal tyrosine followed by glutamate of α-tubulin. Finally, α-tubulin within MTs becomes acetylated, so that the stability and age of a given MT can be judged by several posttranslational modifications^15^. These modifications accumulate during MT aging and in turn contribute to the stabilization of MTs and alter their transport properties. Whereas small molecules such as epothilone B or paclitaxel bind to β-tubulin on the inside of the hollow tubule and directly change the conformation of tubulin disfavoring catastrophe, stabilization of MTs in vivo is also achieved by binding of microtubule binding proteins such as MAP or Tau^16^. Stable MTs with acetylated tubulin and decorated with specific MT binding proteins are a hallmark of healthy axons^17^. In accordance, excitotoxin-induced over-activation of neuronal glutamate receptors causes damage of the microtubule network resulting in axonal breakdown^18^. The stabilization of MT by paclitaxel treatment has been reported to protect axons from fragmentation upon excitotoxin exposure^19^ and it was therefore assigned neuroprotective effects.

Together, axonal degeneration is often found in neurodegenerative conditions such as Alzheimer’s disease^20^, stroke^21^, traumatic brain injury^22^ and Charcot-Marie-Tooth syndrome^23^, and consequently, neurodegenerative diseases are frequently characterized by defective cargo transport along the axon^2,24^. A crucial process for neuronal homeostasis that essentially relies on intact transport routes is neuronal autophagy^25,26^. Whereas the basic molecular machinery of autophagy is highly conserved from yeast to mammalian cells, the spatial separation of autophagosome biogenesis and progression along the axon, and the strong dependence of autophagy on vesicle transport along MT is typical for neurons. In homeostatic neurons, autophagosome biogenesis predominantly occurs in the distal axon^27^. Upon formation, autophagosomes are then transported by the microtubule motor protein dynein on the retrograde route along the axon towards the soma. In agreement with this, neurodegeneration is also characterized by altered autophagic flux. Finally, small molecule drugs such as rapamycin, an immunosuppressant and inducer of autophagy^28^ have been assigned neuroprotective roles^29^ and promise treatment options for human patients, alike the microtubule stabilizing drugs paclitaxel or epothilone B^30^. Notably, the small molecule inducer of autophagy SMER28 was also shown to exert neuroprotective effects in animal models of HD^31^ and AD^32,33^. In recent years, additional cell protective properties of SMER28 have been described on e.g., bone marrow after radiotherapy^34^, or by inhibiting Cytomegalovirus proliferation and cytopathy^35^. The molecular mechanism of SMER28-mediated cell protection, however, has largely remained unknown.

We here set out to better characterize the molecular basis of SMER28 effects on cells. We show that SMER28 treatment in neuronal cells translates into an increased resistance to excitotoxin-induced axon degeneration, confirming its neuroprotective potential. Furthermore, aside from corroborating that SMER28 is a mild inducer of autophagy, we also show that SMER28 significantly alters MT dynamics in cells. Upon treatment, MTs are aligned in a straighter fashion and display increased acetylation. We finally unveil that SMER28 treatment of cells results in decelerated + TIP dynamics, indicating reduced MT polymerization rates. Together, our findings establish that the neuroprotective activity of SMER28 is due to a unique combination of autophagy induction and microtubule stabilization. These two properties are common to known neuroprotective drugs as isolated traits, but emerge here for the first time to be mediated by a single compound.

## Results

### SMER28 pretreatment protects primary neurons against excitotoxin-induced axon degradation

**Figure 1:**
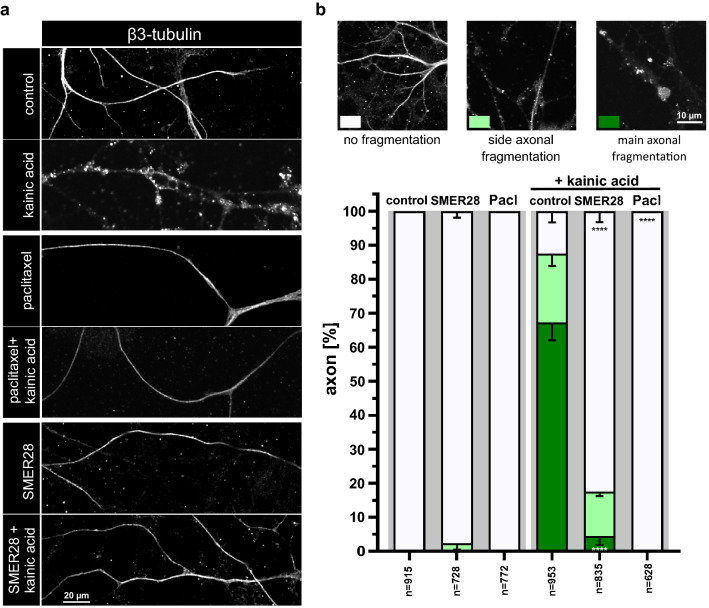
SMER28 protects primary neurons against excitotoxin-induced axon fragmentation. (**a**) Primary neuronal cells were stimulated with DMSO as vehicle control, 150 nM paclitaxel and 50 µM SMER28 in the presence or absence of 25 µM kainic acid for 6 h, as indicated. SMER28 treatment was applied 16 h before kainic acid was added. Cells were stained for β3-tubulin and visualized by spinning disk confocal microscopy. (**b**) Quantification of axon fragmentation. Axons were categorized according to examples shown above the graph as no fragmentation *versus* side axonal fragmentation or main axonal fragmentation. Data were collected form three independent experiments and represent means ± s.e.m; n = total number of axons analyzed. ****P < 0.0001, one way ANOVA.

SMER28 has been identified in a small molecule screen for enhancers of rapamycin and was found to induce autophagy independently of rapamycin’s inhibitory effect on mTORC1, but the mechanism behind this remained unknown^31^. Moreover, SMER28 was described to display significant neuro- and cell protective effects^33,34,36^. We here set out a series of experiments to better understand the cellular and molecular mechanism of the neuro-protective effect of SMER28. We first examined whether SMER28 treatment has the power to protect primary mouse neurons against kainic acid-induced axon fragmentation. Kainic acid is a so-called excitotoxin leading to hyper-activation of glutamate receptors, which in turn leads to axon degradation^37,38^. We isolated neuronal progenitor cells from the subventricular zone of mice not older than 10 weeks and differentiated them into neurons. We next determined the cytotoxicity of SMER28 on these cells and identified 50 µM as the highest tolerable concentration for the duration of our experiments (Supplementary Fig. S1). Next, we induced axonal breakdown by kainic acid treatment in these cells and compared the effect to cells pretreated with either paclitaxel (also known as taxol) as positive control or with SMER28. Paclitaxel is a well characterized stabilizer of MTs and known to antagonize the deteriorating effect of kainic acid^19^. Axonal degradation is a hallmark of many neurodegenerative diseases^17^ and increasing the stability of MTs via drug treatment has been shown to be advantageous in protecting neuronal integrity^39,40^. Axon fragmentation following kainic acid treatment was assessed by immunofluorescence staining for β-III tubulin (Fig. 1a), which is specifically expressed in neuronal cells. Strikingly, SMER28 pretreatment significantly protected axons against degradation by approx. 82% (Fig. 1a,b), to an extent comparable but not as strong as paclitaxel (Fig. 1a middle panel,b).

### SMER28 promotes neurite outgrowth in PC-12 cells

**Figure 2:**
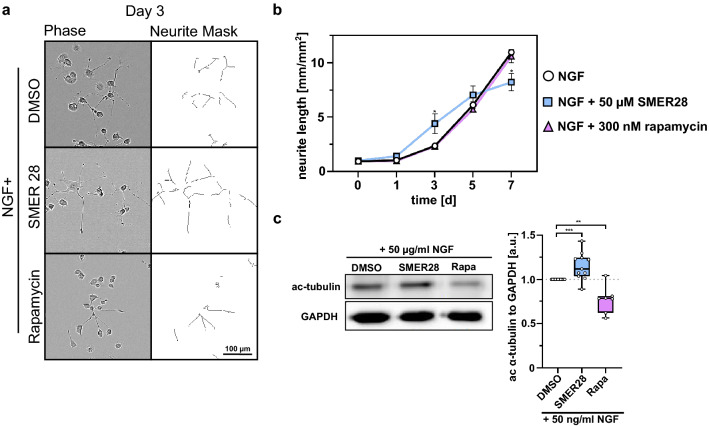
SMER28 promotes neurite outgrowth in PC-12 cells. (**a**) PC-12 cells treated with 50 µg/ml NGF and either DMSO, 50 µM SMER28 or 300 nM Rapamycin were recorded for 7 days. Phase contrast images show cells after 3 days of treatment as indicated on the left panel and neurite outgrowth is depicted on a neurite mask in the right panel. (**b**) Line graph shows the mean neurite length ± s.e.m. from four independent experiments with six replicates. *P < 0.05 (two-tailed t-test). (**c**) Western Blot analysis of PC-12 cells treated with 50 µg/ml NGF in the presence of DMSO, 50 µM SMER28 or 300 nM rapamycin, as indicated, for 3 days. Lysates were analyzed for the protein levels of ac-α-tubulin. GAPDH served as loading control. Quantification of relative ratios of ac-α-tubulin normalized to GAPDH after treatments as indicated. The whiskers of the box and whiskers plots show the min to max of the data. Data from at least seven independent experiments. **P < 0.01, ***P < 0.001, Mann–Whitney rank sum test.

We next turned to PC-12 (rat pheochromocytoma) cells, which embody a well-established model for neurite outgrowth^41,42^, allowing us to determine aspects of neuronal differentiation and regeneration. In these cells, we probed whether SMER28 impacts on the frequency and efficiency of neurite outgrowth. PC-12 cells differentiate into neuronal cells upon nerve growth factor (NGF) stimulation^41^. To investigate the effect of SMER28 in this process, we differentiated PC-12 cells with NGF in the presence or absence of either SMER28 or rapamycin, and assessed the mean neurite lengths (Fig. 2a,b and Supplementary Fig. S2a) or numbers of neurite branch points (Supplementary Fig. S2a,b) by live cell imaging every 24 h over a time period of 7 days. NGF-treated PC-12 cells readily formed branched neurites, a cell response that increased over time. However, in the presence of 50 µM SMER28, neurite formation was significantly increased almost two-fold in the first days but then declined below control levels as assessed by mean neurite length (Fig. 2b). We also noticed increased cell death after 6–7 days in the SMER28-treated condition, likely explaining the observed loss of neurites at later timepoints. Significantly, however, the increase in neurite outgrowth was unique to SMER28, as neurite outgrowth was virtually identical to control in rapamycin-treated PC-12 cells over the entire treatment period (Fig. 2a,b). We next reasoned that based on the neuroprotective features of SMER28, which were reminiscent of MT-stabilizing drugs (compare Fig. 2c), the mechanism of underlying neuroprotection and neurite outgrowth by SMER28 may involve modulation of the MT cytoskeleton. Cytotoxicity of MT-stabilizing, neuroprotective drugs such as epothilone B (EpoB) or paclitaxel (Pacl) exclude application to differentiating cells over longer time periods (Supplementary Fig. S2c) and were thus not analyzed in this assay. However, in order to probe if SMER28 might somehow affect MT-stability, potentially explaining its effects, we chose to examine MT-acetylation. MT acetylation is a post-translational modification and a slow process occurring on α-tubulin only when incorporated into MTs. Increased MT acetylation is associated with enhanced MT stability^43,44^. To test this, we assessed the levels of acetylated tubulin in NGF-differentiated PC-12 cells treated or not with 50 µM SMER28 or rapamycin for three days. Interestingly, MT acetylation levels were indeed moderately increased upon SMER28 treatment, which was not the case for rapamycin (Fig. 2c). Together, this indicates that SMER28 has neurotrophic effects, promoting neurite outgrowth and indicative of a positive role in neuronal differentiation and/or regeneration. Moreover, these experiments revealed that SMER28 results in increased MT acetylation, indicating that it may induce MT stabilization. This prompted us to further analyze the effects of SMER28 on the MT cytoskeleton.

### SMER28 induces formation of straight microtubules

**Figure 3:**
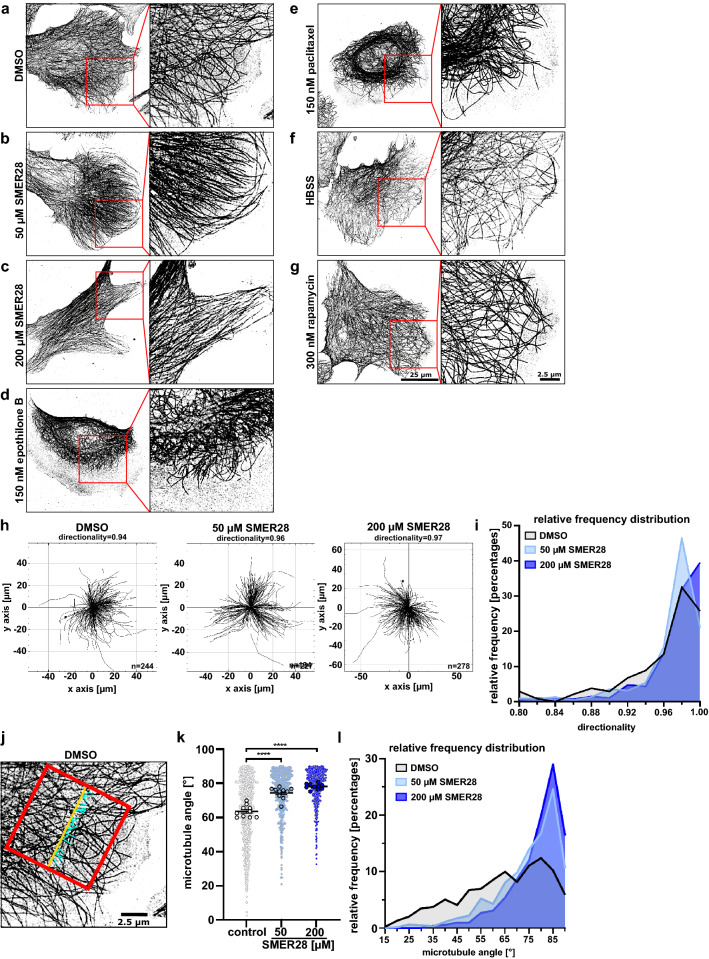
SMER28 induces a straightened arrangement of microtubules. (**a–g**) U-2 OS cells were treated with DMSO as vehicle control, 50 µM or 200 µM SMER28, 150 nM epothilone B, 150 nM paclitaxel, HBSS or 300 nM rapamycin, as indicated, for 4 h. Cells were stained for α-tubulin and visualized by superresolution microscopy, SIM (structured illumination microscopy). Images show maximum intensity projections. (**h**) The Manual Tracking plugin of ImageJ/Fiji was used to manually track and identify microtubule filaments of these images. Microtubule directionality was determined by using the Chemotaxis plugin of ImageJ/Fiji. The trajectory plots show microtubule filaments of DMSO control, 50 µM SMER28 and 200 µM SMER28 treated U-2 OS cells. Quantitative analysis of average directionality of microtubule filaments from at least ten individual cells. n = total number of tracks. (**i**) Relative frequency histogram showing the distribution of microtubule directionality as a measure for their straightness. (**j–l**) Microtubule filament parallelism was analyzed by manual angle measurements using NIS-Elements. (**j**) Exemplary image of a cell edge with reference line (red), drawn parallel to the leading edge to determine the intersecting angles of microtubules (blue). (**k**) Graph depicting all measured microtubule intersecting angles and statistical analysis. Data show means from at least ten individual cells ± s.e.m. ****P < 0.0001, one way ANOVA. (**l**) Relative frequency histogram showing the distribution of intersection angles with 90° relative to the reference line equaling a parallel arrangement of MTs.

To better understand the effects of SMER28 on the microtubule cytoskeleton, we turned to a cell model that displays a widely spread cell shape, namely human osteosarcoma cells (U-2 OS). We treated U-2 OS cells with SMER28 and analysed their morphology, cytoskeletal elements and autophagy markers. We could readily show an increase of LC3-II in treated cells indicative of increased autophagy (Supplementary Fig. S3) as expected^31^ and autophagic flux as recently reported^45^. More importantly, we noted a significant alteration in the appearance of the microtubule cytoskeleton. In SMER28-treated U-2-OS cells, the alignment of MTs was more pronounced and they appeared straighter and more paralleled as compared to the tangled arrangement observed in control cells, as assessed by immunofluorescence staining of α-tubulin (Fig. 3a–c). This straightening effect of SMER28 on MTs was already evident at low concentrations and further increased at higher concentrations, suggesting that MT flexibility decreased with increasing SMER28 concentrations. To probe whether this effect relates to increased MT stabilization, we compared SMER28 to the well-known MT stabilizers epothilone B and paclitaxel. These drugs directly bind to β-tubulin, blocking MT depolymerization^46–48^. However, and in accordance with previous studies, MTs became shorter upon epothilone B- and paclitaxel-treatments, accumulated around the nucleus and no longer extended to the very edges of these cells (Fig. 3d,e)^49^. In contrast, MTs in SMER28-treated cells reached out through the entire cytoplasm, still arriving at the cell periphery (Fig. 3b). We next examined the effect of rapamycin and nutrient withdrawal, both known to induce autophagy, on MT organization, as earlier studies had indicated that also the induction of autophagy might affect microtubule stabilization^50^. However, in line with previous observations^51,52^, we found no obvious differences in subcellular MT patterns upon autophagy induction in U-2 OS cells as compared to control conditions (Fig. 3a,f,g). The striking differences between SMER28-treated MT networks (Fig. 3b,c) and those observed upon treatment with either MT-stabilizing (Fig. 3d,e) or autophagy-inducing conditions (Fig. 3f,g) at concentrations with comparable cytotoxicity^31,45,52,53^ (Supplementary Fig. S2c) implied two aspects: Firstly, our observations indicated that the effects of SMER28-induced MT straightening are distinct from physical MT stabilization as induced e.g. by paclitaxel or epothilone B, and secondly, are not simply due to autophagy induction.

To quantify these effects, we probed the straightness of MTs by assessing the directionality between their ends at the cell periphery back to the cell center, close to the MTOC, inferred from super resolution images of fixed cells stained for microtubules. The measurements (Fig. 3h) revealed that the number of straight microtubules was increased in SMER28 treated cells in a concentration-dependent fashion (Fig. 3h,i). Next, we assessed the parallelism of MTs in the lamella of cells (cytoplasmic region between MTOC and cell front protrusions) by determining the angle of intersection relative to a line through the lamella, perpendicular to the cell front (Fig. 3j,k). Accordingly, significantly more MTs intersected at or close to 90° in SMER28 treated cells (Fig. 3j–l). Together, these quantifications confirm the observation that MTs are straighter and arranged in a more parallel fashion upon SMER28 treatment as compared to control.

### SMER28 treatment leads to increased tubulin acetylation

**Figure 4:**
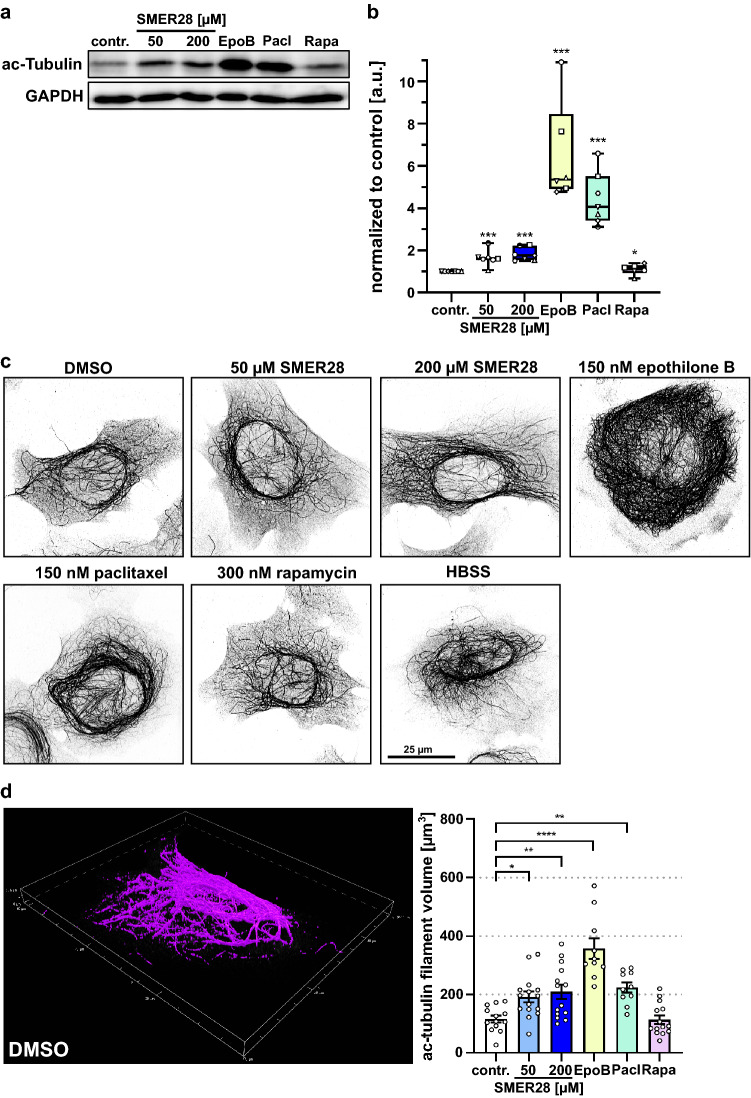
SMER28 treatment stabilizes microtubules by α-tubulin acetylation. (**a**) Western Blot analysis of U-2 OS cells treated with 50 and 200 µM of SMER28, 150 nM epothilone B (EpoB), 150 nM paclitaxel (Pacl) or 300 nM rapamycin (Rapa), as indicated, for 4 h. Lysates were analyzed for the protein levels of acetylated (ac)-α-tubulin. GAPDH served as loading control. (**b**) Quantification of relative ratios of ac-α-tubulin to GAPDH as indicated. Data show means of protein levels derived from six independent experiments ± s.e.m. *P < 0.05, ***P < 0.001, Mann–Whitney rank sum test. (**c**) U-2 OS cells were treated with DMSO as vehicle control, 50 µM or 200 µM SMER28, 150 nM epothilone B, 150 nM paclitaxel, 300 nM rapamycin or HBSS, as indicated, for 4 h. Cells were stained for ac-α-tubulin and visualized by SIM. Images show maximum intensity projections. (**d**) 3D object measurement tool of NIS Elements (Nikon) was used to measure the ac-tubulin filament volume of U-2 OS cells treated as indicated. SIM image stacks were used for quantification. Data show means of ac-tubulin filament volume in µm^3^ from at least ten cells ± s.e.m. *P < 0.05, **P < 0.01, ****P < 0.0001, one way ANOVA.

The MT stabilizing effect of epothilone B and paclitaxel is accompanied by increased acetylation of MTs^46,53^. Increased MT acetylation is in turn associated with enhanced MT stability^43,44^, and inhibition of their deacetylation in turn stabilizes MTs^54^. Thus, we asked if SMER28 also induces MT acetylation like in long term-treated PC-12 cells and if so, whether this relates to paclitaxel or epothilone B treatments or to rapamycin treatment. Interestingly, immunoblot analysis revealed significantly increased levels of acetylated α-tubulin in SMER28-treated samples by approx. 1.7-fold. (Fig. 4a,b). Epothilone B and paclitaxel lead to a strong increase of acetylated α-Tubulin levels by 6.5-fold and 4.5-fold, respectively. Noteworthy, rapamycin treatment did not affect tubulin acetylation. We further confirmed these results by immunofluorescence staining of acetylated α-tubulin (Fig. 4c), and quantified ac-tubulin levels in these cells to make sure that the increased amount of ac-tubulin is actually incorporated into MTs and justifies our claim that SMER28 effects MT acetylation (Fig. 4d). Together, the levels of MT acetylation caused by SMER28 treatment were significant, but less pronounced than those accumulating upon epothilone B or paclitaxel treatment at comparable cytotoxicity (Supplementary Fig. S2c), underscoring once more that the mechanisms of action of SMER28 are distinct from direct, physical MT stabilization. Furthermore, induction of autophagy by rapamycin did not increase levels of acetylated-α-tubulin in contrast to SMER28 treatment, highlighting that the unique effect of SMER28 on MT organization must be independent of the moderate induction of autophagy. To confirm this, we utilized cells genetically lacking ATG5, a core component of the autophagy machinery and thus incapable of recycling amino acids through autophagy^55^. In fact, straight microtubules could also be observed in SMER28-treated ATG5 KO cells (Supplementary Fig. S4a) and, even more convincingly, these were significantly hyper-acetylated in both ATG5 KO and ATG5 control cells (Supplementary Fig. S4b,c). Collectively, we here show for the first time that SMER28 treatment, previously only known to induce autophagy, not only alters MT arrangements, but also enhances their acetylation, likely by affecting MT stability and dynamics. Moreover, we provide evidence that this is not directly due to autophagy induction.

### SMER28 reduces microtubule polymerization dynamics

**Figure 5:**
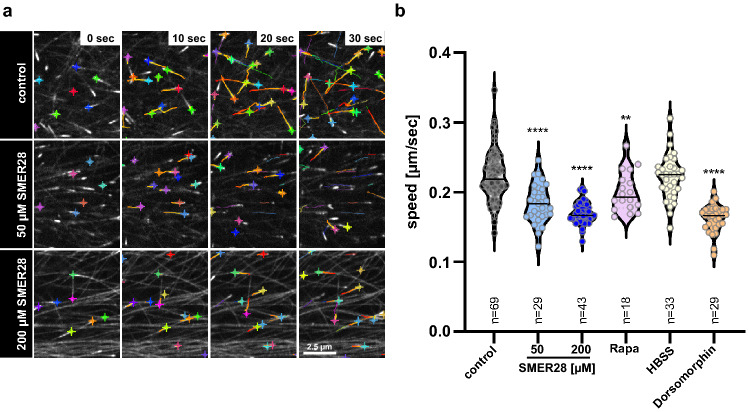
SMER28 reduces microtubule polymerization dynamics. (**a**) U-2 OS cells expressing pEGFP-CLIP-170 were treated as indicated and at a frame rate of 1/sec recorded by spinning disk confocal microscopy 4 h later. Images depict CLIP-170 decorated microtubule plus-ends and tracks of microtubule plus ends detected for a time period of 30 s, indicated by colored lines. Crosses highlight MT plus end positions of respective frames. (**b**) Quantifications from at least three independent experiments are displayed as violin plots, with median; n = total number of videos analyzed; **P < 0.01, ****P < 0.0001 (one-way ANOVA).

Given that MTs are aligned in a straighter fashion and become significantly acetylated upon SMER28 treatment, we asked whether their polymerization dynamics might be altered. Thus, we next explored MT polymerization dynamics in SMER28-treated cells. We utilized live-cell imaging of U-2 OS cells expressing EGFP-tagged CLIP-170, a well-established MT plus-end binding protein that specifically associates at the growing MT-plus-end as part of the + TIP-complex of polymerizing MTs^56,57^. It has been recognized that the speed of + TIP translocation corresponds to the speed of MT assembly^56,58^. We thus determined the speed of GFP-CLIP-170-decorated, MT plus-ends by video microscopy (Fig. 5a), and performed software-assisted velocity analyses of thousands of MT plus-ends (Fig. 5b). These experiments revealed that SMER28 treatment significantly reduces the speed of MT plus-end polymerization in a dose-dependent manner by 19% at 50 µM and by 27% at 200 µM, as compared to DMSO control (Fig. 5a,b, Supplementary movie 1). Subsequently, we probed if also rapamycin might affect the speed of MT plus-end polymerization (Supplementary Fig. S5a). In fact, rapamycin treatment also reduced CLIP-170 speed by 12% in U-2 OS cells (Fig. 5b, Supplementary Fig. 5a). This is in line with the earlier finding showing that rapamycin reduced spindle elongation dynamics in yeast *S. cerevisiae*^59^. Noteworthy, metabolic stress-induced autophagy in HBSS-starved U-2 OS cells did not reduce CLIP-170 speed (Fig. 5b, Supplementary Fig. S5a), indicating that autophagy alone does not account for this effect. In addition to the reduced MT assembly speed in SMER28-treated cells, we observed an increased association of pEGFP-CLIP-170 along MTs at higher concentrations of SMER28 (200 µM), apparent as long comet tails (Supplementary movie 1). CLIP-170 affinity for microtubule tips is believed to be regulated by phosphorylation and to affect in turn MT assembly speed. One prominent kinase that is known to phosphorylate CLIP-170 and to regulate MT assembly is AMPK^13^. To test if an inhibition of AMPK and thus CLIP-170 phosphorylation might display a similar effect, we assessed MT assembly speed by CLIP-170 tracking in the presence of dorsomorphin (also known as compound C), a specific AMPK inhibitor. Of note, this compound was also described to have neuroprotective effects^60^. Inhibition of AMPK was found to control MT assembly speed through phosphorylation of CLIP-170 on Ser311^12^. Strikingly, dorsomorphin treatment reduced MT plus end polymerization speed in amounts comparable to SMER28 (Fig. 5b, Supplementary movie 2, Supplementary Fig. S5a). Since the effects of SMER28 on MT + TIP-velocity were reminiscent of those attained with dorsomorphin, we probed whether SMER28 might also affect CLIP-170-phosphorylation on Ser311. In fact, Western Blotting using pS311-specific antibodies to CLIP-170 revealed that SMER28 also leads to a reduction in CLIP-170 phosphorylation, again virtually identical to dorsomorphin (Supplementary Fig. S5b, c). Together, our results indicate that SMER28-mediated reduction of MT polymerization rates is accompanied by reduced CLIP-170 phosphorylation, reminiscent of the effects seen upon dorsomorphin treatment^12^ (Supplementary Fig. S5).

## Discussion

We here reveal that treatment of cells with SMER28 increases the stability and acetylation of MTs in different cell types like human osteosarcoma U-2 OS cells or rat pheochromocytoma PC-12 cells. Moreover, SMER28 treatment protected the axons of primary neurons from kainic acid-induced axonal breakdown. SMER28 was originally described as an autophagy inducing drug acting independently of mTORC1 inhibition^31^. The process of autophagy is essential for the clearance of protein aggregates, and its deregulation plays a critical role in many diseases such as cancer, infections and neurodegenerative diseases such as Alzheimer’s, Parkinson’s and Huntingtin’s diseases^61^. Consequently, application of SMER28 was already early tested and then shown to promote the clearance of mutant huntingtin protein^31^, and Aβ and APP-CTF^33^.

In neurons, due to their post-mitotic nature and size with long axons, autophagy is not only a temporally regulated sequence of events, but also regulated in a spatial fashion. Autophagosomes in neurons are formed predominantly in the distal tip of the axon and subsequently undergo retrograde transport toward the cell soma via MTs, facilitated by the motor protein dynein. As they move along the axon, autophagosomes mature, progressively fuse with lysosomes and acidify to form the autolysosomal compartment^2^. Therefore, and in addition to derailed autophagy^62^, neuronal cells are particularly susceptible to MT cytoskeleton dysregulation affecting transport^63,64^. Axonal degradation with disturbed MT cytoskeleton is observed in many neurodegenerative diseases, such as Charcot-Marie-Tooth syndrome, affecting MT-stability and eventually axon integrity^65^. Based on these findings, the most promising yet sill experimental approaches to the treatment of neurodegenerative diseases comprise the induction of autophagy with compounds like rapamycin and lithium^66,67^, treatment with small molecules that induce stabilization of MTs like paclitaxel or epothilone B^68^ and, in more recent years, with compounds that induce MT-acetylation via e.g. deacetylase-inhibitors like trichostatin-A and HDAC6 inhibitors^69,70^. Some of these drugs have entered clinical trials, testing their neuroprotective capabilities^29^. For example, the clinically relevant, microtubule-stabilizing agent TPI-287 is currently being investigated for its potential protective functions in tauopathies^71^. Together, stabilization of MTs by induced acetylation^72,73^ as well as induction of autophagy^74^ were all implicated in mediating neuroprotection and are thus the preferred targets of novel treatment approaches^75^.

All these processes are intimately connected: Induction of autophagy has been described to increase MT acetylation and stabilization^50^, and increased MT acetylation upon spermidine treatment has been shown to enhance autophagy^76^. Or bottom up: neurodegenerative diseases are connected to disturbed MT arrangements in the axon^77^, to dysregulated MT acetylation^78^ and to derailed autophagy^61^. Therefore, it is reasonable to assume these are all aspects of one process, namely the spatial coordination of neuronal autophagy, which is disturbed during axon degeneration. The link between autophagy induction and MT stabilization and -acetylation is significant, as in axons, the autophagosomes are moved along stable MTs, ultimately facilitating their maturation to autolysosomes^79,80^. Noteworthy, drugs such as epothilone B and paclitaxel, strongly increasing MT stability and acetylation^46,47^, display a high cytotoxicity in addition to their neuroprotective effects. These in part disadvantageous effects might be due to the fact that they completely block MT dynamics, leading to MT breakage and autophagy inhibition^81^.

In contrast, SMER28 shows a unique spectrum of actions promoting both, autophagy and MT stabilization/acetylation, albeit to a milder degree (Fig. 4). Interestingly, rapamycin, which specifically represses mTORC1 thereby inducing autophagy, did not increase tubulin acetylation (Fig. 4), implying that MT acetylation upon SMER28 treatment is independent of mTORC1 inhibition. Our data therefore confirm that SMER28 acts independently of rapamycin-induced autophagy and thus independent or upstream of mTORC1^31^. Our data also revealed that MT arrangement is more ordered, MT acetylation is increased and MT assembly is slowed down, as exemplified by a significantly reduced speed of + TIPs at MT plus ends. Earlier reports have shown that the speed of MT assembly and of CLIP-170 at MT plus ends is regulated by the phosphorylation of CLIP-170^10–12^. A recent report from our laboratory suggests that the molecular target of SMER28 might be class I PI3 kinases^45^. It is tempting to speculate, therefore, that SMER28 might specifically interfere with a cellular kinase involved in CLIP-170 phosphorylation^12,13,82^, and induces autophagy at the level of or downstream of PI3Ks^83^. PI3Ks are important pharmacologic targets, and drugs modulating their activities are under intense investigation^84–86^. Of note, AMPK is a key energy sensor and regulates cellular metabolism and autophagy induction^87^. Based on our data, we propose a model, in which SMER28 induces autophagy and suppresses CLIP-170 phosphorylation, thereby reducing MT dynamics. Furthermore, our results demonstrate that alteration of MT dynamics is separable from autophagy induction.

Together, we hypothesize that this jointly leads to its neurotrophic and neuroprotective capacity. However, we currently cannot formally exclude the possibility of SMER28 directly modulating the activities of cellular acetyl-transferases or deacetylases. In summary, we show in this study that SMER28, an autophagy-inducing compound, greatly augments MT stability along with decreased MT polymerization rate and increased MT acetylation. In addition, through a combination of live-cell microscopy and immunofluorescence imaging, we show its neurotrophic and neuroprotective capacities. Therefore, the combination of autophagy induction and MT modulation by SMER28 along with an acceptably low toxicity results in a superior neuroprotective effect and makes SMER28 a promising lead structure for the development of new drugs in the treatment of neuropathies.

## Materials and methods

### cDNA cloning

Full length human CLIP-170 was amplified from template cDNA (a kind gift from Dr. Niels Galjart, Erasmus Medical Center Rotterdam, the Netherlands) using forward (CAGAATTCATGAGTATGCTAAAAGCC) and reverse (AGGGATCCTCAGAAGGTTTCGTC) primers and cloned into pEGFP-C2 plasmid (Clontech) using EcoRI and BamHI restriction enzyme sites. The generated plasmid was sequence-verified.

### Animals

Wildtype C57BL/6 mice were housed and handled under license number 325.1.53/56.1-HZI (City of Braunschweig, Veterinary Department) in accordance with good animal practice as defined by FELASA (Federation of European Laboratory Animal Science Associations), the national animal welfare body GV-SOLAS and the institutional animal welfare body, and reported according to the ARRIVE guidelines. Male and female mice aged 6–14 weeks were sacrificed via CO2 inhalation to harvest organs and cells for primary tissue culture in accordance to annex VI directive 2010/63/EU and in compliance with §4 of the German animal protection law (TierSchG BGBI S. 1308; 28.07.2014). All animals used were documented and notified to LAVES (Niedersächsisches Landesamt für Verbraucherschutz und Lebensmittelsicherheit) according to the German laboratory animal reporting act (VersTierMeldV BGBl S. 4145; 12.12.2013)**.** All procedures were in compliance with local regulations and approved by the local animal welfare body.

### Cultivation of cells and transfections

Neuronal progenitor cells were isolated from the sub-ventricular zone of sacrificed mice as described by Walker and Kempermann^88^ and cultured in neuron-medium (Neurobasal, 1 × B27 Supplement, 1 × P/S (Penicillin–Streptomycin), 2 mM l-glutamine and 2 µg/ml heparin) containing 20 ng/ml EGF and 20 ng/ml FGF. Mouse brains were dissected and stored in neuron-medium. Single cell suspensions were plated on cell culture plates pre-coated with poly-d-lysin and laminin^88^. Neuronal progenitor cells were cultured up to 10 weeks after isolation. PC-12 rat pheochromocytoma cells (ATCC CRL-1721) were grown in RPMI-1640 supplemented with 10% heat-inactivated horse serum (Cytogen), 5% FBS (Sigma), 2 mM l-glutamine and 1 × P/S. Differentiation of PC-12 cells was induced by incubation with RPMI-1640 containing 1% heat-inactivated horse serum, 2 mM l-glutamine and 1 × P/S. Cells were incubated at 37 °C in a humidified, 7.5% CO_2_-atmosphere. U-2 OS human osteosarcoma cells (ATCC HTB-96) were cultured in DMEM (4.5 g/l glucose) supplemented with 10% FBS (Sigma), 2 mM l-glutamine, 1 mM sodium pyruvate and 1% non-essential amino acids. Atg5^(+/+)^ MEF (RRID:CVCL_0J74) and Atg5^(−/−)^ MEF (RRID:CVCL_0J75) were kindly provided by Noboru Mizushima^55^ and cultured in DMEM (4.5 g/l glucose) supplemented with 10% FBS (Sigma-Aldrich). U-2 OS cells were transfected with X-tremeGENE 9 DNA Transfection Reagent (Roche) according to manufacturer’s instructions.

### MTT cell cytotoxicity assay

MTT (Sigma-Aldrich) was dissolved at a concentration of 5 mg/ml in PBS, sterile-filtered and stored at − 20 °C. U-2 OS cells were seeded into 96 well plates at a density of 10,000 cells per well and incubated overnight. Cells were treated for 24 h in growth medium containing DMSO as control, 50 µM and 200 µM SMER28 (Sigma-Aldrich), 300 nM rapamycin (Cayman Chemicals), 150 nM paclitaxel (StressMarq Biosciences) and epothilone B (a kind gift from Marc Stadler, Helmholtz Centre for Infection Research, Braunschweig). Treatments were replaced with 100 µl of MTT solution (0.5 mg/ml MTT in DMEM) and incubated for 3 h. The absorbance was read at a wavelength of 590 nm after further 15 min of incubation with 150 µL of MTT solvent (4 mM HCl, 0.1% NP-40).

### Immunofluorescence

For immunolabellings shown in Figs. 3 and 4, U-2 OS cells were seeded onto fibronectin-coated coverslips and processed essentially as described^89^. Briefly, cells were fixed with pre-warmed 4% PFA (paraformaldehyde) containing 0.1% glutaraldehyde in PBS for 20 min and extracted with 0.1% Triton X-100 in PBS for 1 min. Primary antibodies were α-tubulin (clone DM1A, T9026, Sigma-Aldrich, 1:300 dilution, AB_477579) and acetylated α-tubulin (6-11B-1, T6793, Sigma-Aldrich, 1:2000 dilution, AB_477585). Neuronal progenitor cells shown in Fig. 1 were plated onto pre-coated coverslips as described above, and treated for 6 h with neuron medium containing DMSO as control, 25 µM kainic acid (Sigma-Aldrich, K0250), 50 µM SMER28, 150 nM paclitaxel or combinations of these, as indicated. SMER28 was added 16 h prior to the treatment with kainic acid. Neuronal progenitors were fixed with 4% PFA containing 0.25% glutaraldehyde in PBS for 20 min, permeabilized with 0.1% Triton X-100 in PBS for 1 min and stained with β-3-Tubulin (#302302, Synaptic Systems, 1:300 dilution, AB_10637424) antibodies. Cells were mounted in ProLong Diamond Antifade (Invitrogen).

### Live-cell imaging

U-2 OS cells transiently expressing pEGFP-C2-CLIP-170 were seeded into a 4 well µ-slide (Ibidi) and imaged by confocal spinning disk microscopy at 37 °C in a humidified, 7.5% CO_2_ atmosphere. Data were collected at 1 Hz for approximately one minute. PC-12 cells were imaged in a 96 well plate using an IncuCyte S3 Live-Cell Analysis System (EssenBioscience) with a 10 × objective at 37 °C and a humidified 5% CO_2_ atmosphere. The differentiation medium containing the indicated treatment was added 6 h after cell seeding.

### Microscope set-ups

Data shown in Figs. 1 and 5 were acquired on a Zyla 4.2 sCMOS camera (Andor) with a CSU-W1 spinning disk (Yokogawa) mounted on a Ti2 eclipse microscope (Nikon). The Apo Plan 100 × oil/NA1.4 and 60 × oil/NA1.4 objectives and a 405/488/561/638 nm laser (Omicron) were used in combination with an Okolab stage top incubation chamber (Okolab) in case of live-cell experiments. For SIM (3D structured illumination microscopy) images shown in Figs. 3 and 4, an N-SIM E (Nikon) was used, built on a Ti-Eclipse microscope (Nikon). Data were acquired using a z Piezo drive (Mad city labs), an Apochromat TIRF 100 × Oil/NA 1.49 objective, an Orca flash 4.0 LT sCMOS camera (Hamamatsu), a LU-N3-SIM 488/561/640 laser unit (Nikon) and a motorized N-SIM quad band filter combined with a single 525/50 emission filter using the laser line 488 at maximum output power. Z-stacks were acquired with a step size of 200 nm. Both microscopes were controlled by NIS-Elements software (Nikon). Slice reconstruction (NIS-Elements, Nikon) was performed using reconstruction parameters IMC 0.7, HNS 0.7, OBS 0.2.

### Western blotting

Cells were washed thrice with ice-cold PBS and lysed with 4 × SDS-laemmli sample buffer. Proteins were separated by SDS-PAGE and analysed by immunoblotting. The primary antibodies GAPDH (clone 6C5, #CB1001, Calbiochem, 1:5000 dilution, AB_1285808), acetylated α-tubulin (6-11B-1, T6793, Sigma-Aldrich, 1:2000 dilution, AB_477585), phospho-CLIP-170 (Ser311) (kindly provided by Yasunori Shintani, National Cerebral and Cardiovascular Center, Osaka, Japan^13^, 1:1000 dilution), CLIP-170 (#8977, Cell Signaling Technology, 1:1000 dilution, AB_11178661) and actin (#A2066, Sigma-Aldrich, 1:5000 dilution, AB_476693) were detected by using Lumi-Light Western Blotting Substrate (Roche) and the ECL Chemocam Imager (Intas). For densitometric analysis, bands were normalized to the levels of GAPDH. For the simultaneous detection of acetylated tubulin (55 kDa) and GAPDH (36 kDa), membranes were cut at 45 kDa and for the simultaneous detection of LC3 and GAPDH, membranes were cut at 20 kDa. Membrane sections were incubated with the respective antibodies and imaged. Uncropped raw images of the Western Blots used can be found in the appendix to the Supplementary Information.

### Data processing and statistical analyses

Image analysis was carried out using Fiji (https://imagej.net/software/fiji/), IncuCyte software (Essen BioScience) and NIS-Elements (Nikon). Neurite outgrowth was analyzed with the IncuCyte NeuroTrack Software Module. Mean neurite length and mean neurite branch points were measured over the time course of 7 days. Microtubule tracking was performed with the NIS-Elements Analysis Advanced 2D Tracking module (Nikon). Tracks containing more than 10 frames were taken into account. Further data processing was carried out in NIS-Elements (Nikon), Fiji/ImageJ, Inkscape (Inkscape Project), Prism 9 (Graphpad) and Excel 2010. Statistical tests, sample sizes and numbers of experiments are given in the figure legends.

## Supplementary Information

Supplementary Information 1.

Supplementary Movie 1.

Supplementary Movie 2.

## Data Availability

All data generated or analyzed during this study are included in this published article and its supplementary information files.
