# Supplementary material for: The autophagy inducer SMER28 attenuates microtubule dynamics mediating neuroprotection
Source: Sci Rep. 2022 Oct 25;12:17805. doi: 10.1038/s41598-022-20563-3 (PMC9596692; doi:10.1038/s41598-022-20563-3)

Supplementary Information

Supplementary Figure S1

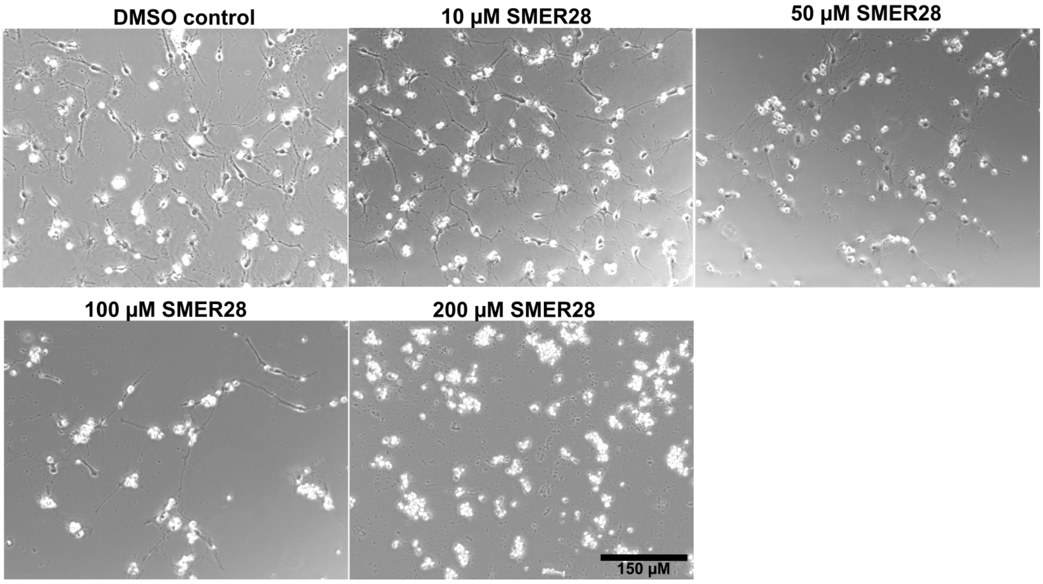

Supplementary Figure S1**: Different SMER28 concentrations on differentiated primary neurons.** *In vitro* differentiated primary neuronal cells were treated with DMSO as vehicle control and with SMER28 (10 µM, 50 µM, 100 µM, 200 µM) for 16h. Phase contrast images show cells after 16h treatment.

Supplementary Figure S2

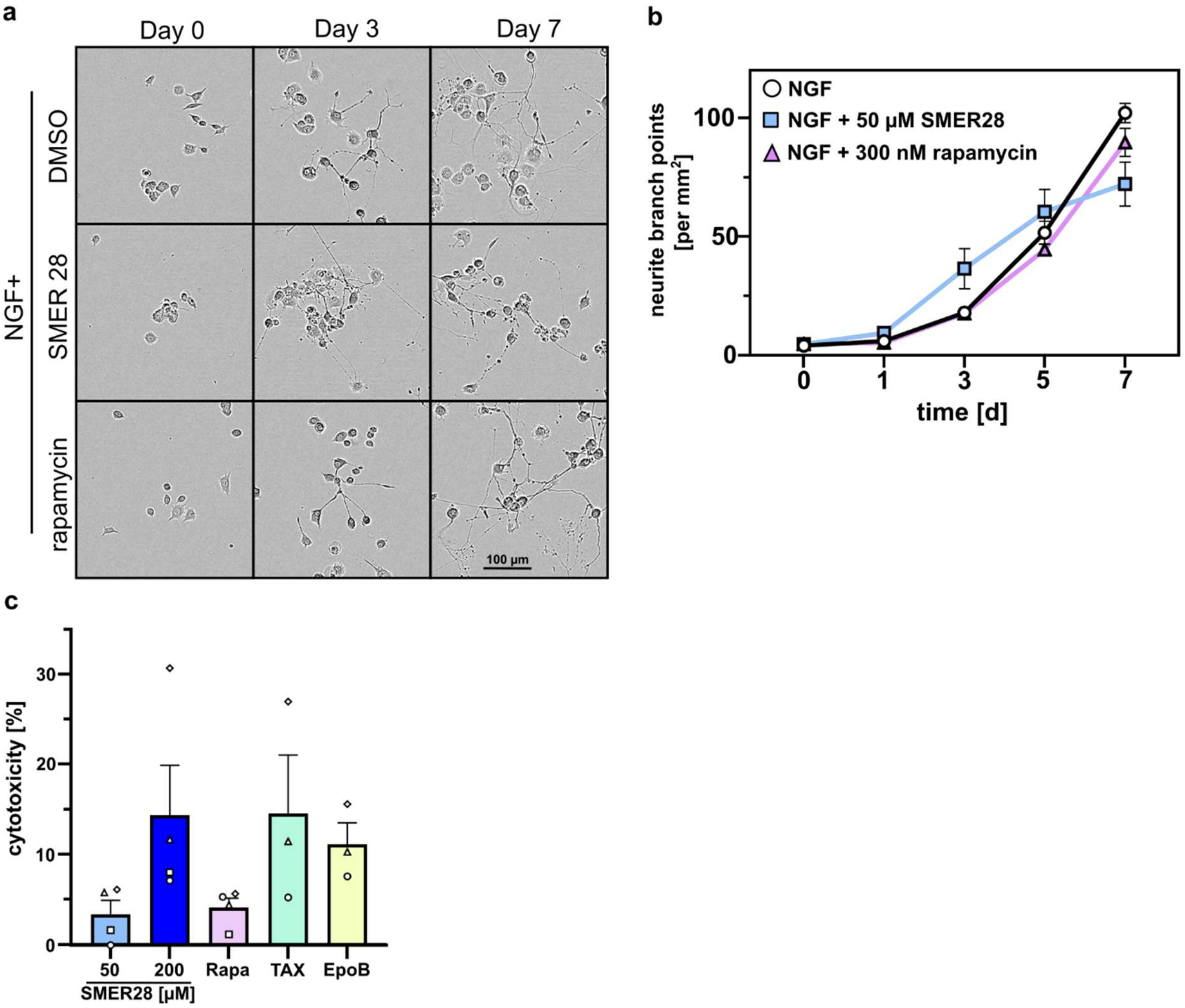

Supplementary Figure S2: **SMER28 induces mild cytotoxicity and promotes neurite outgrowth in PC-12 cells. (a)** PC-12 cells were treated with 50 µg/ml NGF and 50 µM SMER28, 300 nM Rapamycin or DMSO control and recorded by phase contrast imaging. Phase contrast images depict neurite outgrowth after 0-, 3- and 7-days. **(b)** Graph shows the mean neurite branch points ±s.e.m. from four independent experiments with six replicates. **(c)** MTT assay of 50 µM or 200 µM SMER28, 300 nM rapamycin, 150 nM epothilone B or 150 nM paclitaxel in comparison to DMSO as vehicle control in U-2 OS cells. Graph shows the mean cytotoxicity ±s.e.m. from three independent experiments. *P<0.05 (two-tailed t-test).

Supplementary Figure S3

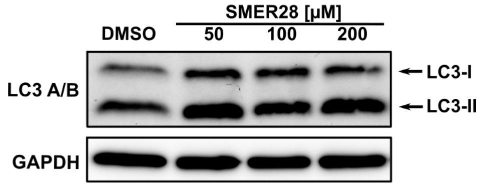

Supplementary Figure S3**: SMER28 induces LC3 production in different concentrations.** U-2 OS cells treated for 16 hours with different concentrations of SMER28, as indicated, or DMSO control were analyzed by Western blot with LC3 antibodies or GAPDH as loading control.

Supplementary Figure S4

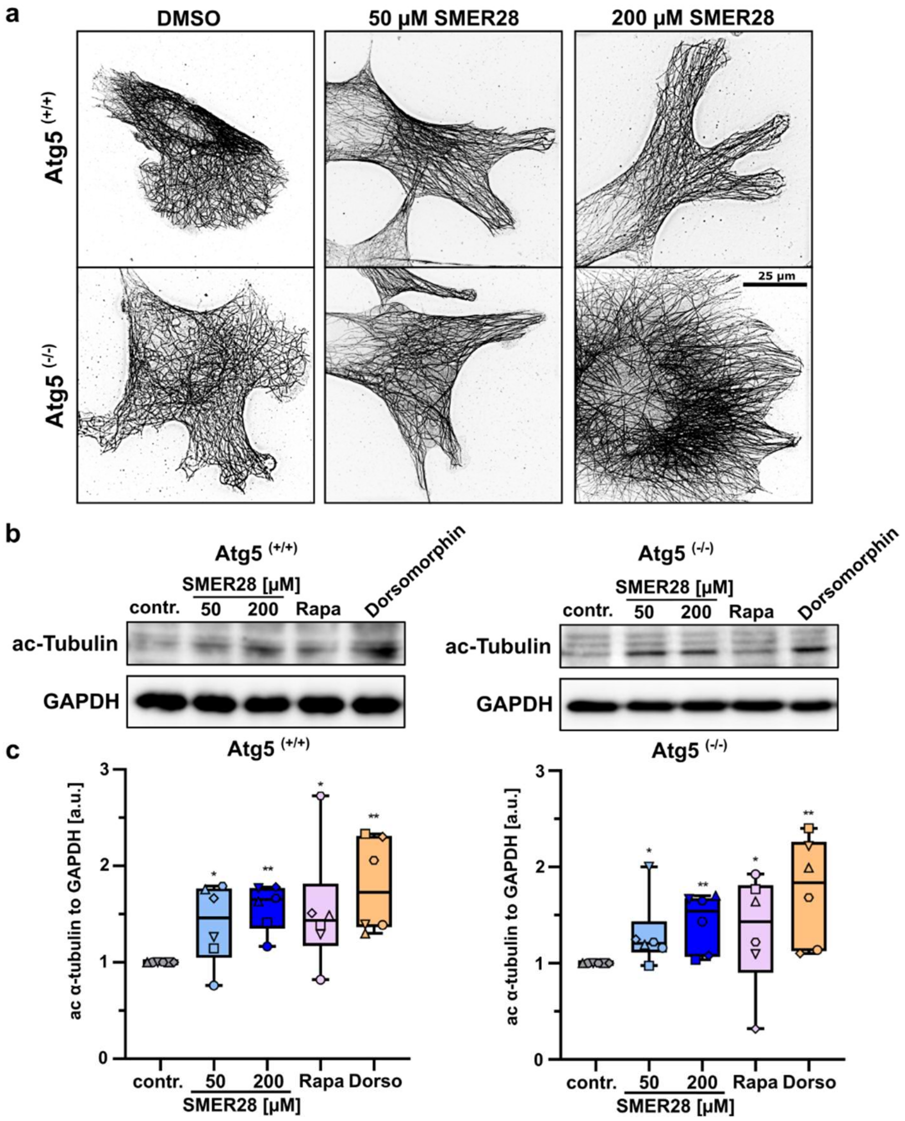

Supplementary Figure S4**: SMER28 alters MT arrangement and -acetylation independent of autophagy. (a)** Atg5$^{(+/+)}$ MEF and Atg5$^{(-/-)}$ MEF cells treated with DMSO as vehicle control, 50 µM SMER28 or 200 µM SMER28, as indicated, for 4h. Cells were stained for α-tubulin and acquired by SIM. **(b)** Western blot analysis of Atg5 $^{(+/+)}$ MEF and Atg5 $^{(-/-)}$ MEF were treated with DMSO as vehicle control, 50 µM SMER28, 200 µM SMER28, 300 nM rapamycin or 4 µM dorsomorphin, as indicated, for 4h. Lysates were analyzed for the protein levels of acetylated (ac) α-tubulin. GAPDH served as loading control. **(c)** Quantification of relative ratios of ac-α-tubulin to GAPDH as indicated. Data show box & whiskers plots with whiskers from min to max of protein levels derived from at least six independent experiments. *p<0.05, **p<0.01, ***p<0.001, Mann-Whitney rank sum test.

Supplementary Figure S5

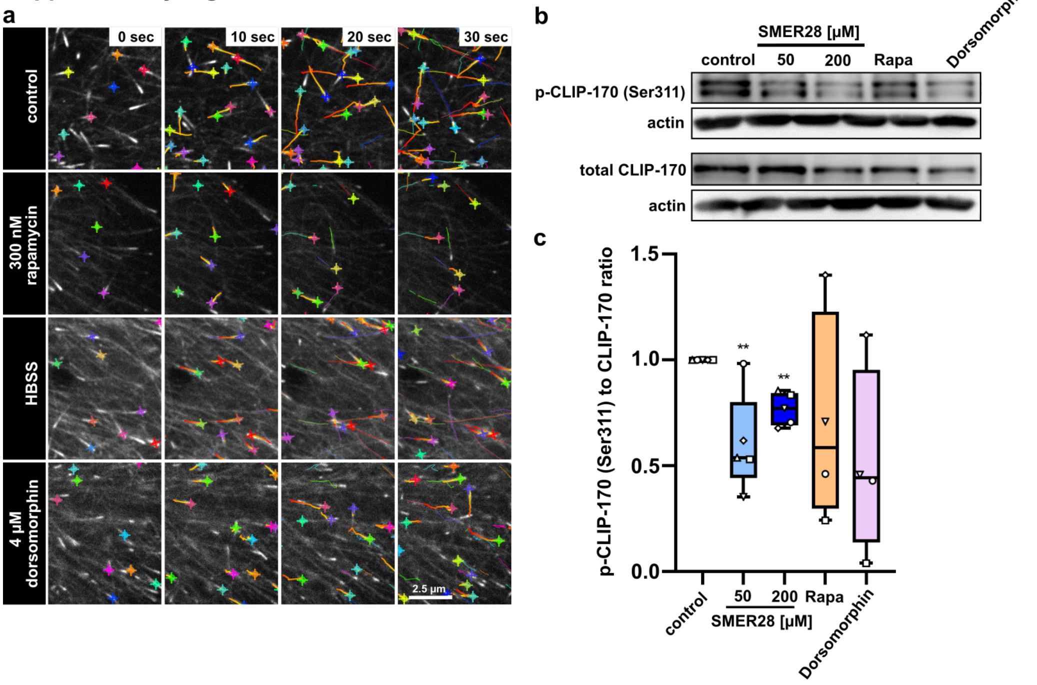

Supplementary Figure S5: **EGFP-CLIP-170 dynamics after treatment with rapamycin, HBSS or dorsomorphin**; related to Fig. 5b. **(a)** Images show EGFP-CLIP-170 dynamics expressed in U-2 OS cells after treatments and time points as indicated. Color crosses highlight MT plus-ends and color trails indicate passed tracks. **(b)** Western Blot analysis of U-2 OS cells treated with 50 and 200 µM of SMER28, 300 nM rapamycin (Rapa) or 4 µM dorsomorphin, as indicated, for 4h. Lysates were analyzed for the protein levels of phospho-CLIP-170 (Ser311) and total CLIP-170. Actin served as loading control. **(c)** Quantification of relative ratios of phosphorylated CLIP-170 to total CLIP-170 normalized to actin. Data show box & whiskers plots with whiskers from min to max of protein levels derived from at least four independent experiments. **$p<0.01$, Mann-Whitney rank sum test.

Supplementary Movie S1. U-2 OS cells expressing pEGFP-CLIP-170 were treated with 50 µM or 200 µM SMER28 for 4 hours, as indicated. Cells were imaged every second for 1 minute by spinning disk microscopy. Scale bar: 100 µm.

Supplementary Movie S2. U-2 OS cells expressing pEGFP-CLIP-170 were treated with 300 nM rapamycin, HBSS or 4 µM dorsomorphin for 4 hours, as indicated. Cells were imaged every second for 1 minute by spinning disk microscopy. Scale bar: 100 µm.

APPENDIX

Uncropped raw Western Blot images

Raw images to Figure 2c

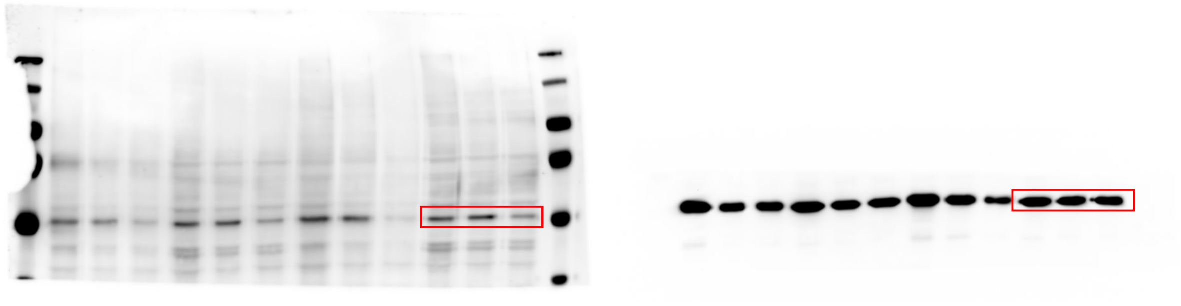

Raw images to Figure 4a

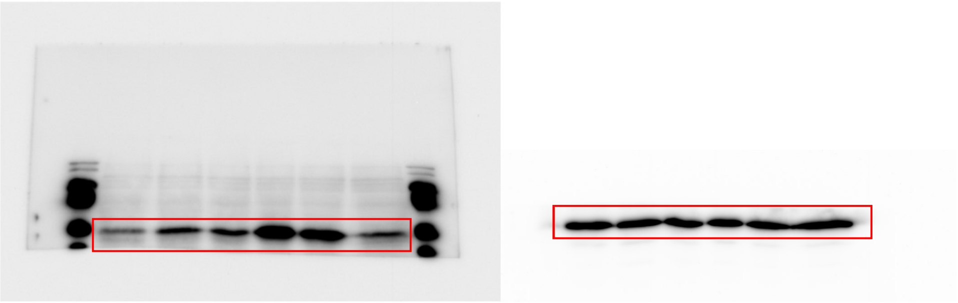

Raw images to Supplementary Figure S3

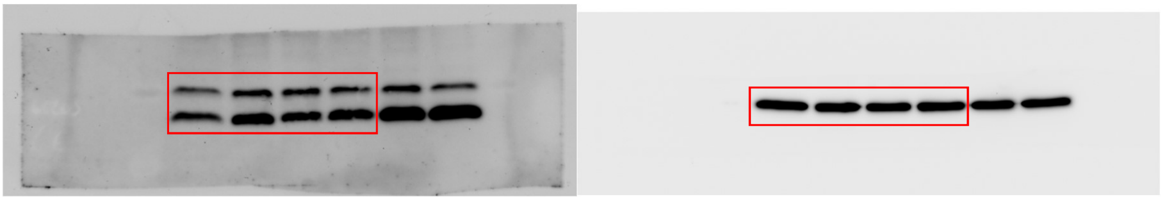

Raw images to Supplementary Figure S4b

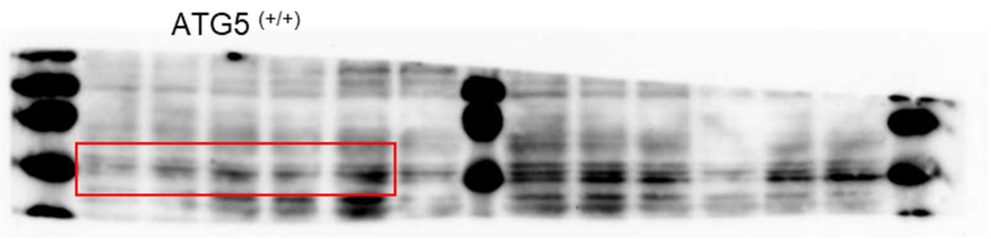

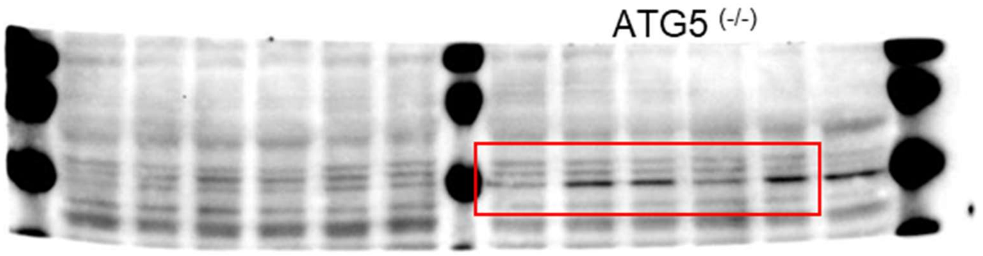

Raw images to Supplementary Figure S5c

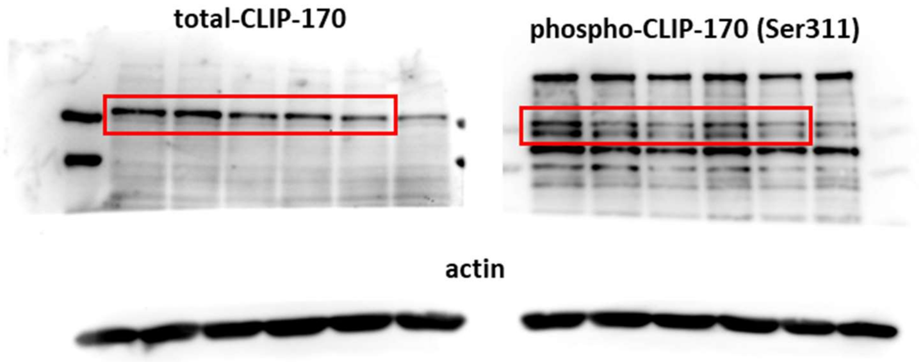

Supplement: Supplementary file 1 — Supplementary Information 1. [file 41598_2022_20563_MOESM1_ESM.pdf]
